# Impact of mask wearing on cultivable conjunctival bacteria in Chinese healthcare workers

**DOI:** 10.1038/s41598-026-51290-8

**Published:** 2026-05-06

**Authors:** Wenwen Wei, Yanli Shi, Qingjun Hu

**Affiliations:** 1 Department of Ophthalmology of Civil Aviation General Hospital, Civil Aviation Medical College of Peking University, Beijing, 100123 China; 2 Department of Clinical Laboratory of Civil Aviation General Hospital, Civil Aviation Medical College of Peking University, Beijing, 100123 China

**Keywords:** Mask wearing, Ocular surface, Microbiota, Healthcare workers, Diseases, Medical research, Microbiology

## Abstract

This study investigated the temporal impact of mask wearing on cultivable conjunctival bacteria among healthcare workers. Conjunctival samples were collected from 48 participants, who were randomly assigned to wear surgical masks or KN95 respirators. Sampling occurred at baseline (T0, no mask), after 8 h (T1), and after one week of daily wear (T2, ≥ 8 h/day). Microbial cultures were analyzed using Matrix-Assisted Laser Desorption Ionization Time-of-Flight Mass Spectrometry. The viable bacteria were of low diversity, dominated by Firmicutes, Actinobacteria, and Proteobacteria, encompassing seven families. Staphylococcaceae, Bacillaceae, and Corynebacteriaceae were most prevalent. In the KN95 group, Staphylococcaceae relative abundance was significantly higher at T2 than at T1 (*P* = 0.009). In the surgical mask group, Corynebacteriaceae abundance was significantly higher at T2 versus T0 and T1, while Bacillaceae was lower (all *P* < 0.01). Significant differences in Bacillaceae across all timepoints were unique to the KN95 group (all *P* < 0.01). At T1, Staphylococcaceae abundance was higher in the surgical mask group, while Bacillaceae was lower compared to the KN95 group (*P* = 0.025 and *P* = 0.023, respectively). At T2, Corynebacteriaceae abundance was significantly higher in the surgical mask group (*P* = 0.010). These findings raise the possibility that mask wearing may induce subtle, mask-type-dependent alterations in the cultivable conjunctival bacteria. Further study with a control group is warranted.

## Introduction

As we have moved through the post-COVID-19 era, self-consciousness about wearing medical masks to prevent disease has gradually become the new normal^1^. However, as with any intervention, there may be fallout from the widespread use of masks in the general population, especially among individuals who use masks for extended periods of time (the elderly, those with compromised immune systems, and clinic staff)^2^. Since wearing face mask redirects exhaled air upwards to the periorbital area^3^, attention is drawn to ocular surface irritation and symptoms^4,5^.

Mask wearing has been reported to be associated with increased incidence of viral conjunctivitis^6^, chalazion^3^, recurrent corneal erosion syndrome, and corneal infections^7^ during the COVID-19 pandemic. Endophthalmitis is a potentially serious and destructive complication of ophthalmic and intraocular surgeries^8^. And increased risk of endophthalmitis during intravitreal injections has also been reported to be associated with mask wearing in the past years^9–11^. The reason and mechanism of this situation remain unknown.

The potential for airflow from the superior edge of the mask to contaminate multiuse eyedrop bottles during eyedrop administration in eyecare centers has been detected using thermal camera devices^12^. Previous research has shown that the unique airflow when mask wearing directed more bacteria toward the ocular surface than when no mask worn^13^. Nevertheless, the chocolate agar plates utilized in these studies are not a precise model of the ocular surface^8,14^, leading to uncertainty regarding the actual effects of mask-wearing on ocular microbiota.

Based on the above studies, the aim of the current study is to investigate the impact of wearing two kinds of different face masks (surgical mask and KN95 respirator) on cultivable conjunctival bacteria in Chinese healthcare workers by using conventional culture-based methods at three timepoints. Therefore, by elucidating these mechanisms, we propose to offer novel evidence for updating clinical guidelines, with the goal of lowering ocular infection incidence among individuals requiring prolonged mask use.

## Results

This study enrolled 48 participants, from whom 144 conjunctival samples were collected and successfully cultured on chocolate agar plates. Participants were randomly allocated to two groups: 25 to the surgical mask group and 23 to the KN95 respirator group. In the surgical mask group, the mean subject age was 47.48 ± 15.26 years, and 20.00% (5/25) were male. In the KN95 respirator group, the mean age of subjects was 43.00 ± 15.01 years, and 21.74% (5/23) of subjects were male. No significant intergroup difference was observed regarding age or sex distribution (both *P* > 0.05).

### Microbial community analysis via culture-based methods

Using conventional culture-based methods, microorganisms were successfully isolated from 93.06% (134/144) of conjunctival samples. Matrix-Assisted Laser Desorption Ionization Time-of-Flight Mass Spectrometry (MALDI-TOF MS) identified 19 distinct bacterial genera or species. These viable conjunctival bacteria belonged to three dominant phyla: Firmicutes, Actinobacteria, and Proteobacteria, distributed across seven detected families.

**Fig. 1:**
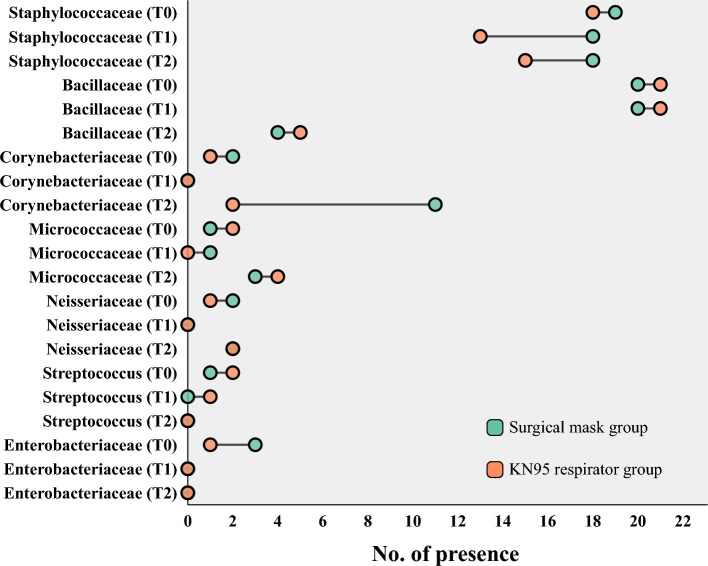
Number of samples of the presence of seven microbiota families in the surgical group and the KN95 respirator group.

**Fig. 2:**
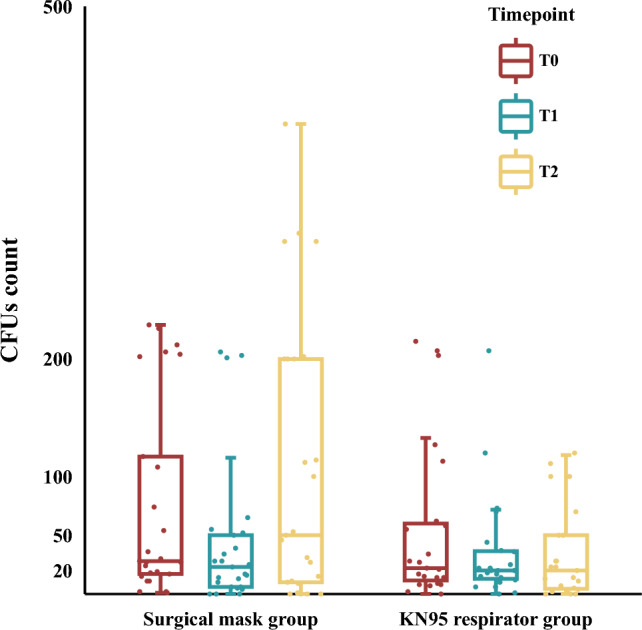
CFU count of the surgical mask and KN95 respirator group at three timepoints. *Notes* T0 (Baseline, no mask), T1: Eight hours after mask wearing, T2: one week of daily mask wear (≥ 8 h/day). No significant intergroup difference was detected at any timepoint (all* P* < 0.05).

Although family distribution varied among individuals, the three most prevalent were Staphylococcaceae (70.14%, 101/144), Bacillaceae (63.19%, 91/144), and Corynebacteriaceae (11.11%, 16/144) (Fig. 1). The taxa most commonly seen also had the highest colony-forming unit (CFU) counts per sample. When present, CFUs ranged from 1 to over 200 per conjunctival sample, with a maximum of four species observed in any single subject. No significant difference in CFU counts was found between the two mask groups at any timepoint (all *P* > 0.05) (Fig. 2). No bacterial species was universally present across all subjects or at all timepoints.

The highest relative abundance of Staphylococcaceae was observed in the surgical mask group at T1, while the lowest occurred in the KN95 respirator group at the same timepoint. Conversely, Bacillaceae reached its peak abundance in the KN95 group at T1 and was lowest in the surgical mask group at T2. For Corynebacteriaceae, abundance was highest in the surgical mask group at T2 and lowest in both groups at T1.

Then, the differences in microbial relative abundance were compared within and between the surgical mask and KN95 respirator groups at all three timepoints at a family level, respectively. Within the surgical mask group, the relative abundance of Staphylococcaceae remained stable across all timepoints (all *P* > 0.05). While in the KN95 respirator group, the relative abundance of Staphylococcaceae was seen significantly higher at T2 compared to T1 (*P* = 0.009), with no difference observed between T0 and T1 or T2 (*P* = 0.044 and *P* = 0.281, respectively). In the surgical mask group, the relative abundance of Bacillaceae was significantly lower at T2 than at T0 and T1, respectively (both *P* = 0.000), while no difference was observed between T0 and T1 (*P* = 0.149). While in the KN95 respirator group, significant differences were observed in all three timepoints (all *P* < 0.01). In the surgical mask group, the relative abundance of Corynebacteriaceae was significantly higher at T2 than T0 and T1 (*P* = 0.008 and *P* = 0.003, respectively), while no difference was found between T0 and T1 (*P* > 0.05). In the KN95 respirator group, the relative abundance of Corynebacteriaceae remained stable across all timepoints (all *P* > 0.05).

**Fig. 3:**
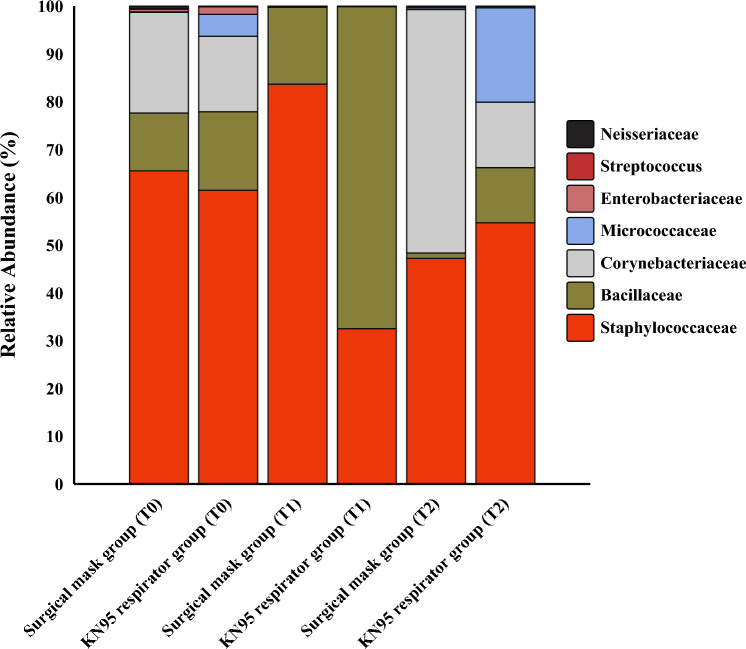
Relative abundance of seven microbiota families in the surgical group and the KN95 respirator group over time. *Notes* T0 (Baseline, no mask), T1: Eight hours after mask wearing, T2: one week of daily mask wear (≥ 8 h/day). Microbiota abundance at the family level was compared within and between mask groups over time (T0, T1, T2). Staphylococcaceae increased at T0/T2 vs. T1 in KN95 only. Bacillaceae decreased at T2 in surgical masks; all timepoints differed in KN95. Corynebacteriaceae increased at T2 in surgical masks only. Between groups: at T1, surgical masks had higher Staphylococcaceae and lower Bacillaceae; at T2, surgical masks had higher Corynebacteriaceae (all *P* < 0.05). No baseline differences.

At baseline, no significant intergroup differences in microbial abundance were observed for any family (all *P* > 0.05). At T1, the surgical mask group exhibited higher Staphylococcaceae abundance, but lower Bacillaceae abundance compared to the KN95 respirator group (*P* = 0.025 and *P* = 0.023, respectively), while after adjustment, no significant difference was observed. By T2, the abundance of Corynebacteriaceae was significantly greater in the surgical mask group than in the KN95 group (*P* = 0.010) (Fig. 3). No significant differences, either between groups or across timepoints within groups, were detected for Micrococcaceae, Neisseriaceae, Streptococcus, or Enterobacteriaceae (all *P* > 0.05). All the cultivable families showing statistically significant intergroup differences were common constituents of the ocular surface microbiota.

*Staphylococcus epidermidis* (43.75%, 21/48 subjects) and *Brevibacillus* (14.58%, 7/48 subjects) were present in the same individuals across all timepoints, demonstrating a degree of longitudinal taxonomic stability at the individual level.

**Fig. 4:**
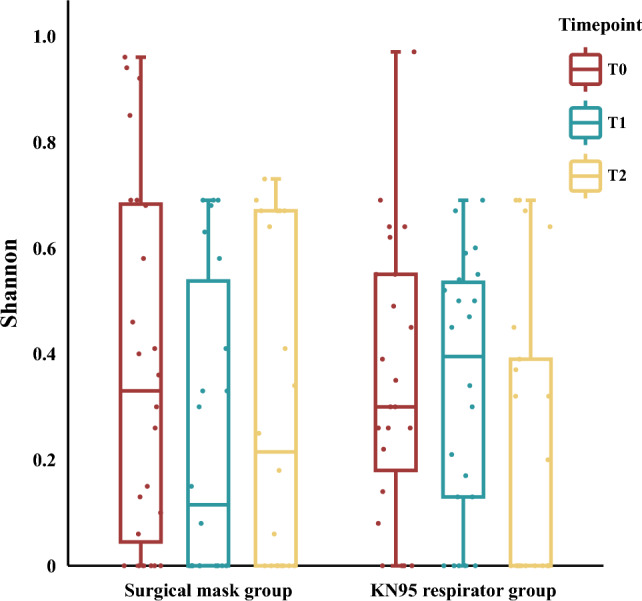
Shannon’s diversity index of the surgical group and the KN95 respirator group over time. *Notes* No significant intergroup difference was detected at any timepoint (*P* > 0.05).

Shannon’s diversity index was used to assess the diversity of cultivable conjunctival bacteria, with no significant difference observed between the two groups over time (all *P* > 0.05) (Fig. 4).

## Discussion

The current study examined the effects of wearing two kinds of face masks (surgical masks and KN95 respirators) on cultivable conjunctival bacteria in healthcare workers. We investigated whether there was any difference in viable conjunctival bacteria changes between before and after wearing mask by using conventional culture-based methods at three timepoints. Understanding whether conjunctival bacteria vary along with wearing mask may shed light on whether wearing mask could be related to the high incidence of inflammatory ocular disorders.

To our knowledge, this represents the first investigation into the impact of face masks on cultivable conjunctival bacteria in a Chinese healthcare worker cohort. Given the limitations of the cultivation methods, all analyses in the present study were confined to these cultivable conjunctival bacteria. Consistent with existing literature^15,16^, the dominant phyla identified were Firmicutes, Actinobacteria, and Proteobacteria. We detected a total of nineteen bacterial genera or species to be present on the ocular surface, belonging to seven families. Previous studies have indicated that Propionibacterium is a common bacterium found on facial and ocular surfaces^17,18^. However, it was not detected in our investigation. A possible explanation is that Propionibacterium typically requires an extended incubation period of 5–10 days for observable growth^19,20^, whereas our protocol employed a shorter culture duration of only 48 h. Interestingly, although detected at low abundance, we successfully identified several bacterial species uncommon on the ocular surface, such as *Rothia dentocariosa*, a bacterium typically colonizing the oral cavity and oropharyngeal mucosa^21^. We speculate that there may be some possible transfer from its typical habitat in the mouth and throat after wearing mask. Further studies analyzing nasal swab samples could elucidate potential correlations between the nasal and ocular microbiomes to clarify whether mask wearing may induce or facilitate the transfer of oral and pharyngeal bacteria, thereby potentially altering the ocular surface microbial environment.

Due to the low abundance of microorganisms in the conjunctival samples, to guarantee sufficient statistical power, we compared the differences within and between the two groups at all timepoints at a family level. Culture results suggested that the three most common bacterial families (Staphylococcaceae, Bacillaceae, and Corynebacteriaceae) showed some uneven variation in both groups over time. Notably, the relative abundance of Staphylococcaceae and Bacillaceae showed the greatest variation at T1 (8 h after mask wearing) but returned to levels with no significant intergroup difference at T2 (one week later). This pattern indicated a short-term impact and subsequent normalization of the cultivable conjunctival bacteria. The situation was quite different when comparing the relative abundance of Corynebacteriaceae. The highest relative abundance of Corynebacteriaceae was identified at T2 in the surgical mask group; significant intergroup variation was observed at the same timepoint. The balance of commensal bacteria on the ocular surface is jointly maintained by the potent antimicrobial properties of the tear film and the mechanical cleansing action of the eyelids^22,23^. Thus, we deduce that to some degree, the balance is sufficient in a short period of time, while it seems quite fragile in a relatively long period of time when wearing mask is taken into consideration. However, further studies are required to validate the relevant hypotheses. Ozkan et al.^24^ investigated the effects of face mask on the ocular surface (conjunctiva, eyelid margin, and contact lens surfaces) microbiome using both culture and culture-independent methods and found that wearing mask did not significantly alter the overall microbiome structure of the healthy ocular surface, only slightly increased the isolation rate of *Staphylococcus capitis* on contact lenses.

A higher Shannon’s index value indicates greater bacterial community diversity^15^. Predictably, the diversity of ocular surface microbiota is commonly low according to previous studies^25^. But surprisingly, in our study, Shannon’s diversity index was conspicuously lower. We infer that the possible reason for the difference is the different subjects recruited. In the current study, subjects are healthcare workers from the same hospital.

Owing to occupational requirements, healthcare workers frequently wear face masks during their routine working hours. It is commonly assumed that mask wearing, which redirects exhaled air towards the ocular surface, is associated with increased tear evaporation and compromised tear-film quality^26^. Tears play a key role as natural barriers against pathogenic bacteria in the ocular surface^5^. In this context, healthcare workers are at a disadvantage. To minimize confounding factors and improve the comparability of research outcomes, participants were uniformly required to have no mask worn for at least 48 h prior to enrollment in the current study.

Notwithstanding, the present study has several important limitations. First, the resolution of the VITEK MS system is limited for certain genera (e.g., *Brevibacillus*), preventing full species-level identification of all isolates. Second, under the given experimental conditions, non-culturable organisms (e.g., anaerobic bacteria) might not have been optimally cultivated and, therefore, may have been underrepresented in the results. We acknowledge that incorporating metagenomic or 16S rRNA sequencing would allow characterization of non-culturable organisms and provide a more comprehensive view of the microbial community. We intend to explore it in future work. Third, all participants were recruited from a single hospital, resulting in relatively homogeneous samples. Fourth, although our sample size exceeded the minimum suggested for validation studies^24,27^, it remained insufficient to detect differences in rare bacterial taxa. Finally, due to local public health mandates requiring universal mask wearing during the study period, we were unable to include control groups (e.g., non-healthcare workers or healthcare workers without masks). We acknowledge that without a control group or crossover design, it is difficult to definitively distinguish the observed changes in conjunctival bacterial detection from normal temporal variability, such as seasonal fluctuations, environmental factors, or other time-dependent variables unrelated to mask wearing. Therefore, the lack of a control group necessitates caution in drawing causal inferences. Specifically, the inclusion of a control group would have provided critical information in the following areas: 1) Baseline variability: It would have helped quantify the natural fluctuations in conjunctival bacteria over time in the absence of any intervention; 2) Causal attribution: It would have allowed for a more robust attribution of any observed alterations in bacterial detection to mask use rather than to external environmental or temporal factors; 3) Confounding control: It would have helped mitigate the influence of confounding variables such as seasonal changes, humidity, or variations in personal hygiene practices. We suggest that future studies employ randomized controlled or crossover designs to more accurately assess the impact of mask wearing on the ocular surface microbiome.

In summary, our results reveal slight changes in the common bacteria of the conjunctiva associated with the two different kinds of mask usage. Given that no significant changes were detected in CFU counts or Shannon diversity, these findings should be interpreted with caution. They raise the possibility, rather than provide definitive evidence, that mask wearing may induce subtle, mask-type-dependent alterations in the cultivable conjunctival bacteria. To enhance the validity of these findings, it is of paramount importance to incorporate a non-mask control group. Furthermore, future studies with larger sample sizes and more heterogeneous populations are justified.

## Methods

### Inclusion/Exclusion criteria

This study was performed in compliance with the Declaration of Helsinki and adhered to the National Regulations for the Ethics of Biomedical Research Involving Human Subjects issued by the Chinese Ministry of Health. All experimental protocols were approved by the Ethics Board of Civil Aviation General Hospital, Civil Aviation Medical College of Peking University (Approval No.: 2022-L-K-57), and informed study consent was obtained from all participants. The inclusion criteria involved: (1) Age over 18 years, capable of cooperating with Microbiota sampling; (2) Willing and able to wear the uniformly provided masks as required by the study protocol; (3) No mask worn for at least 48 h prior to enrollment. Exclusion criteria: (1) Previous corneal refractive or intraocular surgery; (2) Presence of ocular surface diseases such as keratitis, corneal trauma, corneal ulcer, keratoconus, corneal leukoma or macula, congenital corneal anomalies, blepharitis, or pterygium; (3) History of corneal contact lens wear; (4) Use of any ocular surface medication within the past month; (5) Conditions including strabismus, nystagmus, uveitis, glaucoma, or uncooperative behavior during examinations; (6) Uncooperative behavior or inability to attend scheduled examinations as required.

### Interventions

Participants were randomly assigned to wear either a three-layer non-woven fabric surgical mask (Zhende Medical Supplies Co., China) or a KN95 respirator (3 M Company, equivalent to the N95 standard). All masks were provided by the investigators, and participants were instructed on the correct wearing technique to ensure an airtight seal. To reduce confounding factors as much as possible, subjects were told to maintain their work and lifestyle rhythm as usual. Participants were instructed to cleanse their faces exclusively with lukewarm water^1^. To minimize the risk of mechanically removing or altering the microbial flora on the periocular skin—which could be caused by washing with water or cleansers—and to ensure standardization of sampling conditions across all participants, face washing was not permitted in the 12 h preceding microbial sampling during the formal study period^15^.

Three sampling timepoints were established to assess the temporal effects of mask wearing on the cultivable conjunctival bacteria. Baseline (T0, no mask): Participants arrived at a totally separate appointed room in the early morning of the initial enrollment day, conjunctival sampling was then taken by a designated proficient operator and used as the baseline reference. Eight hours after mask wearing in the afternoon of the same day (T1, mask wearing except water access and lunch period) and one week later with mask wearing over 8 h per day (T2): Sampling was conducted again by the same operator following the same protocol, respectively.

### Microbiota sampling and culturing

Samples were collected at T0, T1, and T2 from lower lid conjunctival sacs of both eyes (10 s each side) of all subjects using sterile cotton swabs (BIORISE, Langbin Company, China). Swabs were randomized between eyes to control for order effects. Samples from both eyes of the same subject were pooled to culture and analyze. The culturing procedure was conducted immediately at the Department of Clinical Laboratory, Civil Aviation General Hospital, Civil Aviation Medical College of Peking University in Beijing, China. Chocolate agar plates incubated at 37 °C with 5% CO₂ were used as the standard culture condition to support reliable growth of all bacterial strains^28^. The bacterial culture conditions of the present study were as follows: chocolate agar plates (Columbia type, 90 mm diameter, Thermo Fisher Scientific Biochemicals Company, China), incubation at 37 °C in a 5% CO₂ environment for 48 h. To standardize organism recovery, plates stored at 4 °C were first equilibrated to room temperature prior to use^13^. Bacterial colony-forming units (CFUs) were quantified, and species were identified by microbiologists blinded to the plate collection sequence^14^.

### Microbiota identification

The MALDI-TOF MS system (Model: VITEK MS, bioMérieux Company, UK) was applied in the identification of viable microorganisms^29^. The detailed identification protocol was as follows^30^:(1) Sample Preparation: Isolated bacterial colonies from pure culture plates were transferred onto a target slide (32 slides × 48 wells/box, bioMérieux, France); (2) Matrix Application: Each sample spot received 1 µL of α-cyano-4-hydroxycinnamic acid (HCCA) matrix solution (5 × 0.5 ml, bioMérieux, France) and was air-dried; (3) Instrument Analysis: The target slide was then inserted into the VITEK MS instrument; 4) Data Processing And Identification: The acquired mass spectral profile of the unknown isolate was compared in real-time against the integrated reference database (VITEK MS SARAMIS™ database), organism identification was automatically generated by the system software with a corresponding confidence value.

### Statistical analysis

SPSS 21.0 (IBM Company, USA) was utilized for statistical analysis. Mean ± standard deviation depicted normally distributed data (e.g., age) and were assessed through Student’s t-test. Categorical data (e.g., sex) were presented as proportions and analyzed with the Chi-Square test. Non-normally distributed data (e.g., relative abundance) were described as median and interquartile range, and analyzed using non-parametric techniques. The Friedman test was conducted to detect differences across the three timepoints within each group. When the Friedman test yielded a significant result, Dunn’s post-hoc test was employed to identify which specific pairs of time points differed from each other. Intergroup differences were analyzed using the Mann–Whitney U test. To ensure adequate statistical power, within-group and between-group comparisons of relative abundance were conducted at the family level across all timepoints, with significance defined as *α* = 0.05. *P*-values were calculated using the Wilcoxon rank-sum test, with correction for multiple testing performed via the Bonferroni method (*α’* = *α* / N, N = times for comparisons). *P* < *α* means a statistically significant difference. All figures shown were drawn using ChiPlot (https://www.chiplot.online/).

## Data Availability

All data generated or analyzed in this study are included in this published article. Additional datasets are available from the corresponding author upon reasonable request.
